# Poster Session II – Poster of Distinction II - A207 PRIMARY HUMAN FECAL MUC2 ADMINISTRATION AMELIORATES COLITIS, DYSBIOSIS, AND MUCUS DEFECTS WITHIN A COLITOGENIC HUMANIZED INTESTINAL ECOSYSTEM IN MICE

**DOI:** 10.1093/jcag/gwaf042.207

**Published:** 2026-02-13

**Authors:** D Kniffen, M Dirks, E Howard, P Daneshgar, W Zandberg, K Bergstrom

**Affiliations:** Biology, The University of British Columbia Okanagan, Kelowna, BC, Canada; Biology, The University of British Columbia Okanagan, Kelowna, BC, Canada; Biology, The University of British Columbia Okanagan, Kelowna, BC, Canada; Biology, The University of British Columbia Okanagan, Kelowna, BC, Canada; Biology, The University of British Columbia Okanagan, Kelowna, BC, Canada; Biology, The University of British Columbia Okanagan, Kelowna, BC, Canada

## Abstract

**Background:**

Densely O-glycosylated mucus is a crucial mediator of barrier function and protection from microbially induced colitis; however its therapeutic potential, particularly in a human-relevant context, is unclear. This is partly due to the difficulty in accessing large quantities of in vivo produced natural (primary) human MUC2, the major protein in mucus. Recent insights showing fecal association and encapsulation of microbial boli have enabled non-invasive access to primary human MUC2 for functional analysis.

**Aims:**

We tested the hypothesis that primary human fecal MUC2 can act as a prebiotic to restore homeostasis in a preclinical model of colitis induced by defective mucus within a humanized colonic ecosystem.

**Methods:**

A humanized colitogenic ecosystem was generated using TM-inducible epithelial-specific deletion of core 1 synthase (C1galt1f/f;VilCreERT2) to produce underglycosylated mucus with a human-like core 3 O-glycan profile, followed by colonization with human microbiota on a human-profile diet (Hu:TM-IEC C1galt1-/-). After colitis establishment (2 weeks post-TM), exogenous fecal MUC2 naturally enriched in core 3 O-glycans was administered. Clinical disease activity, histopathology, in situ mucus structure, 16S rRNA sequencing, and inflammatory gene expression (qPCR, ELISA) were assessed.

**Results:**

Prior to induction, Hu-TM-IEC C1galt1-/- mice retained a robust mucus layer that was rapidly degraded post-TM, leading to spontaneous colitis and validating the model. Human MUC2 treatment reduced clinical disease activity by restoring body weight and decreasing diarrhea. Histology confirmed reduced hyperplasia and inflammatory infiltrates. RT-qPCR and ELISA showed decreased IL-6, IL-23a, and Ccl2 expression in colonic mucosa post-treatment. Mechanistically, MUC2 restored the endogenous proximal colon derived barrier layer, indicating protective effects in microbiota-adapted proximal regions. 16S profiling revealed increased representation of eubiotic-associated taxa and elevated anti-inflammatory short-chain fatty acids (acetic and propionic acid) in MUC2-treated mice.

**Conclusions:**

Primary human fecal MUC2 ameliorates colitis by restoring endogenous mucus barrier function and promoting eubiosis. This is the first demonstration of therapeutic application of exogenous human MUC2 in a humanized intestinal ecosystem.

**Funding Agencies:**

Weston Family Foundation

